# Dynamic analysis of CSF1R-related leukoencephalopathy on magnetic resonance imaging: a case report

**DOI:** 10.1186/s12883-021-02182-z

**Published:** 2021-04-10

**Authors:** Huasheng Huang, Liming Cao, Hong Chen

**Affiliations:** 1 Department of Neurology, Liuzhou People’s Hospital, Liuzhou, China; 2 Department of Neurology, The First Affiliated Hospital of Shenzhen University, 3002 Sungang West Road, Futian District, Shenzhen City, 518000 China; 3 Department of Neurology, The Third Affiliated Hospital of Shenzhen University, Shenzhen, China

**Keywords:** Colony-stimulating factor 1 receptor, Leukoencephalopathy, Magnetic resonance imaging, Mutation, Case report

## Abstract

**Background:**

Colony-stimulating factor 1 receptor (CSF1R)-related leukoencephalopathy is a rare and rapidly progressive leukoencephalopathy characterized by cognitive, motor, and neuropsychiatric symptoms, which is often misdiagnosed. Magnetic resonance imaging (MRI) signs and follow-up MRI of CSF1R-related leukoencephalopathy could help in establishing a diagnosis, but these features are not widely known by general neurologists.

**Case presentation:**

A 34-year-old man was admitted for progressive weakness of the right limbs over 8 months. His father and sister had a similar clinical evolution. The primary neurological signs were hemiplegia, cognitive decline, dysarthria, pyramidal signs, ataxia and parkinsonism, and rapid disease progression. Cerebrospinal fluid analysis results were normal. Despite receiving treatment for improving cerebral metabolism and relieving the muscle spasm, his symptoms did not improve significantly. Brain MRI showed lesions concentrated in the corpus callosum and the deep white matter of the bilateral parieto-occipital lobes, periventricular areas, and corticospinal tracts. There was an enhanced lesion after a gadolinium-enhanced MRI scan. Over the 8-month progression, the lesions always exhibited restricted diffusion. The diffuse lesions gradually increased as the disease progressed. Genetic sequencing results showed a novel heterozygous missense mutation (c.2267 T > C p.L756P) in the CSF1R gene. The patient was treated with citicoline and idebenone for 4 days to improve cerebral metabolism, but his symptoms did not improve significantly.

**Conclusion:**

The multiple lesions involving the pyramidal tract and white matter showed continuously restricted diffusion on brain imaging and gradually increased with disease progression.

## Background

Colony-stimulating factor 1 receptor (CSF1R)-related leukoencephalopathy is a rare and rapidly progressive leukoencephalopathy characterized by cognitive, motor, and neuropsychiatric symptoms [1, 2]. It is also considered as an autosomal dominant disease. It has been discovered that CSF1R is a causative gene for hereditary diffuse leukoencephalopathy with spheroids (HDLS) [3] and, more recently, for pigmented orthochromatic leukodystrophy [4]. Carriers of CSF1R pathogenic variants develop adult-onset leukoencephalopathy with axonal spheroids and pigmented glia (ALSP) [4]. ALSP can be easily misdiagnosed at the initial examination because the clinical and radiologic presentations are similar to those of other diseases, such as Alzheimer’s disease, multiple sclerosis (MS), or cerebral small vessel diseases. Magnetic resonance imaging (MRI) signs and follow-up MRI of CSF1R-related leukoencephalopathy could help establish a diagnosis and thereby prevent misdiagnosis, but they are not widely known by neurologists. In this paper, we discuss the dynamic analysis of a case of CSF1R-related leukoencephalopathy.

## Case presentation

In April 2020, a 34-year-old man was admitted with an 8-month history of progressive numbness, weakness, and clumsy movements of the right limbs. Initially, right hemiplegia had appeared in August 2019, followed by slurred speech 2 months later. Shortly after the symptoms began, a white matter disease was discovered on brain MRI, MS was diagnosed, and a treatment trial by methylprednisolone pulse therapy was performed. Despite these treatments, his symptoms worsened. His medical history was unremarkable, other than smoking 20 cigarettes a day for 6 years. His family had a relevant medical history. His father developed dementia and epilepsy at around 50 years of age, developed limb palsy, became bedridden, and ultimately died at 55 years of age. Meanwhile, the patient’s sister had experienced cognitive decline (Mini-Mental State Examination = 23/30) at the age of 40 years and was found to have bilateral paraventricular white matter lesions with diffusion restriction on MRI.

Physical examination showed clear consciousness; dysarthria; memory, comprehension, and calculation impairment; dimpled right nasolabial sulcus; dysphagia; slowed pharyngeal reflex; high muscular tension in all extremities (spastic extremities); and decreased muscle strength in the left limbs (4/5), the right proximal limbs (4/5), and the right distal limbs (3/5). Our patient also demonstrated a positive Romberg sign, pain and tactile sensory disturbances in his right limbs, right patellar hyperreflexia, and right positive Babinski sign. His Mini-Mental State Examination score was 14/30, and his water swallow test score was 3/5.

**Fig. 1:**
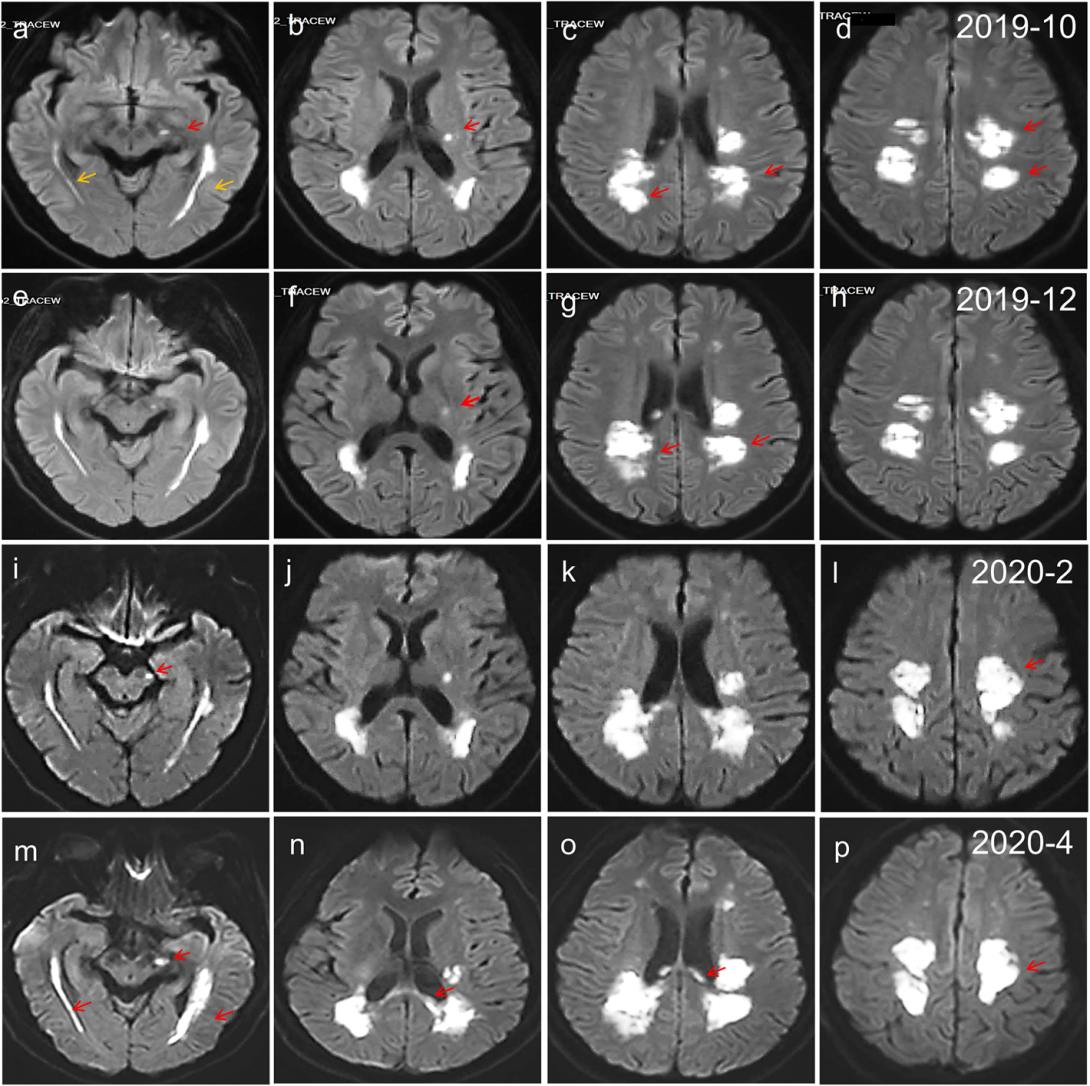
Diffusion-weighted imaging over time. Each row shows diffusion-weighted imaging at different times (from top to bottom: Oct 2019, Dec 2019, Feb 2020, and Apr 2020). The lesions showed restricted diffusion during progression, which lasted for 8 months. The foci were point-like (**a**, **b** red arrows), cord-like (**a** yellow arrows), patchy (**c**, **d** arrows), and incompletely symmetric (**c**, **d**, arrows) in the early stage. Subsequently, the lesions revealed progressive confluency (**g** and **l** arrows), and the lesions gradually enlarged (**l**, **m** arrows) and increased in areas such as the corpus callosum (**n**, **o** arrows)

**Fig. 2:**
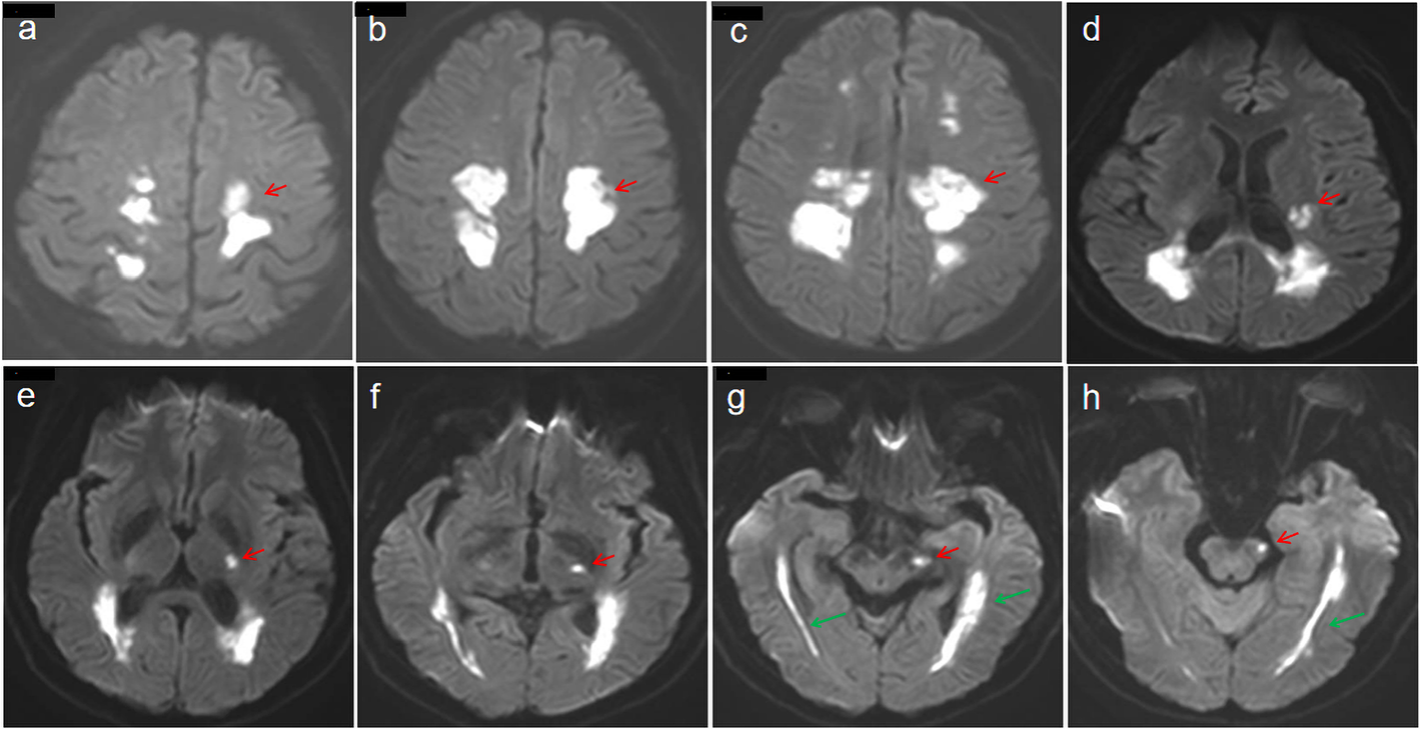
DWI sequences on different levels of the brain in April 2020. The bilateral pyramidal tracts had high signal intensity at different levels (**a**-**h**), especially on the left side (**g**, **h**). The diffusion limitation of the bilateral optic radiation (green arrows) and the corticobulbar tract (red arrows) extends from the cerebral cortex to the midbrain

**Fig. 3:**
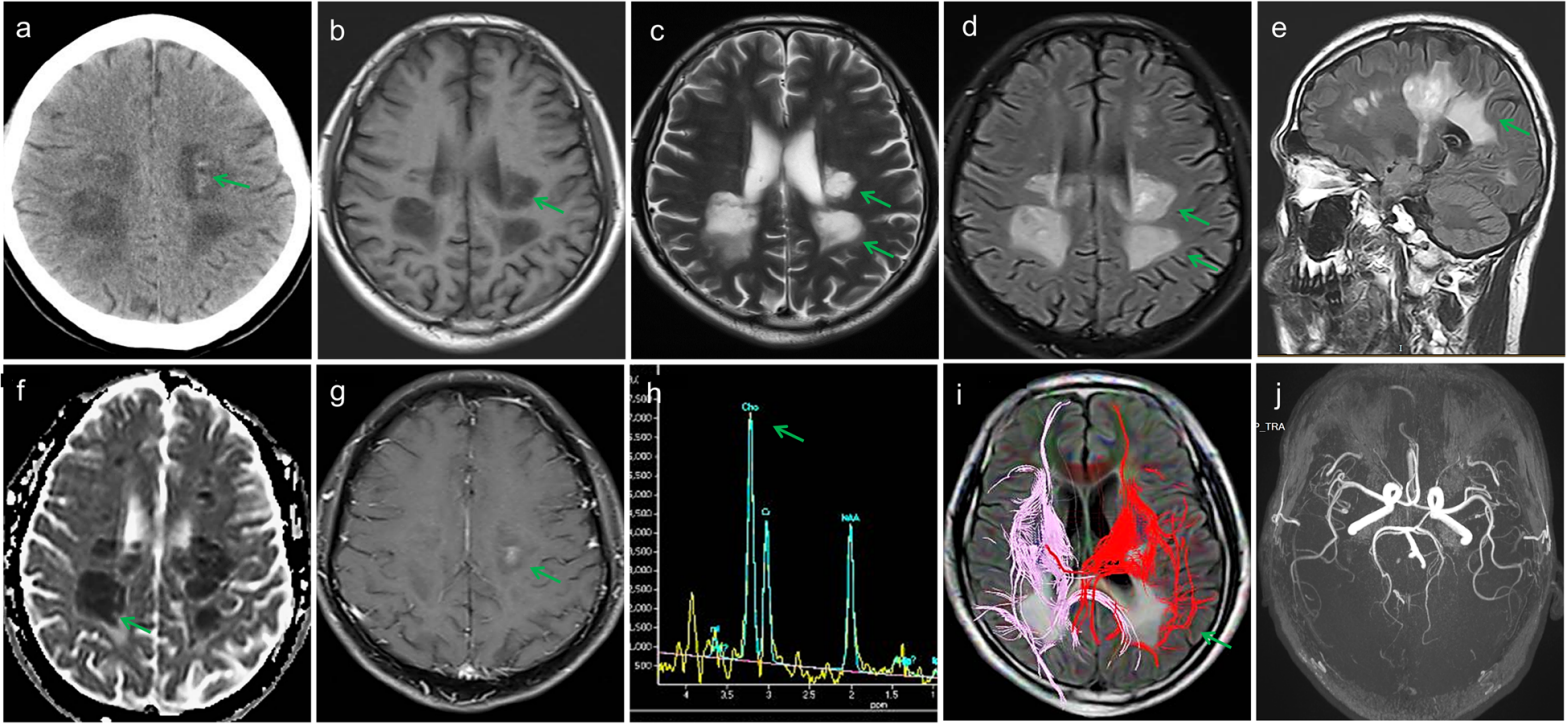
Appearance of HDLS on different image sequences. **a** Computed tomography shows dot high-density shadows within the foci, resembling islands (arrow). **b** Equal signal intensities within the low signal focus on T1 WI (weighted imaging), like islands (arrow). **c** and **d** Axial T2 WI (**c**) and fluid-attenuated inversion recovery imaging (**d**) show patchy high signal focus in the deep white matter of the bilateral parieto-occipital lobes, paraventricular area, and posterior horn (arrows). **e** The midsagittal fluid-attenuated inversion recovery sequence shows obvious involvement of the corpus callosum. **f** Apparent diffusion coefficient shows low signal intensity in the foci. **g** Enhanced magnetic resonance imaging shows non-enhancement of most lesions. **h** Magnetic resonance spectroscopy shows a significantly increased choline wave in the lesions. **i** Diffusion tensor imaging shows sparse fiber conducting bundles within the bilateral subcortices of the parieto-occipital lobes, paraventricular white matter, and corpus callosum. Most of the bundles are absent. **j** Magnetic resonance angiography shows no apparent abnormalities

**Fig. 4:**
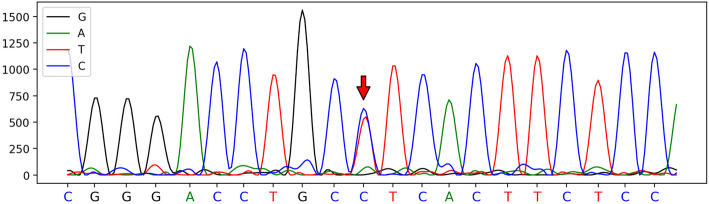
Genetic study findings. The genetic sequencing result indicates an exon 18 heterozygous missense mutation (c.2267 T > C) in the CSF1R gene. The arrow shows the precise position of the pathogenic variation on the sequencing result

Laboratory tests showed elevated total cholesterol (5.87 mmol/L) and triglyceride (1.63 mmol/L) levels. Syphilis, human immunodeficiency virus, rheumatoid factor, anti-double-stranded DNA, anticardiolipin, antineutrophil cytoplasmic, and antinuclear antibody tests were negative. Alpha-fetoprotein (14.36 ng/mL), carbohydrate antigen 72–4 (56.819 U/mL), and blood lactate (2.54 mmo1/L) levels were slightly increased. The initial cerebrospinal fluid pressure was 192 mmH_2_O, and the cerebrospinal fluid analysis results were normal. Brain MRI showed lesions concentrated in the corpus callosum and the deep white matter of the bilateral parieto-occipital lobes, periventricular areas, and corticospinal tracts. There was an enhanced lesion after the gadolinium-enhanced MRI scan (Figs. 1, 2 and 3). Genetic sequencing results showed a novel heterozygous missense mutation (c.2267 T > C p.L756P) in the CSF1R gene (Fig. 4). Further testing showed that the patient’s sister had the same gene mutation. According to the American College of Medical Genetics and Genomics gene mutation guidelines, the mutation was classified as “likely pathogenic.” The mutation was not included in the specialized Human Gene Mutation Database, the Clinvar database, or the gnomAD database. The patient was diagnosed with CSF1R-related leukoencephalopathy. The patient was treated with citicoline and idebenone for 4 days to improve cerebral metabolism. Additionally, he received baclofen and gabapentin for muscle spasms, but his symptoms did not improve significantly. Nevertheless, he was satisfied with the treatment. On a visit 8 months after his discharge, he was bedridden without working ability and had epilepsy and visual impairment.

## Discussion and conclusions

Our patient definitely met the diagnostic criteria [1] for ALSP, and we identified a novel CSF1R mutation in this case. For 8 months, our patient presented many lesions that showed early-stage restricted diffusion, and the lesions gradually increased. However, our patient had no frontal lobe involvement, ventricular enlargement, or cortical atrophy, which was inconsistent with the previous report [5]. Our patient’s computed tomography (CT) scan showed a dot high-density shadow within the foci, which we speculated to be calcifications.

As CSF1R is a crucial mediator of microglial proliferation and differentiation in the brain, CSF1R-related leukoencephalopathy is regarded as a primary microgliopathy. Since the discovery of CSF1R gene mutations in families with HDLS in 2012, over 70 different mutations have been identified worldwide. Typically, patients present with a frontotemporal dementia-like phenotype in their 40s or 50s accompanied by motor symptoms, including pyramidal and extrapyramidal signs. Women develop clinical symptoms at a younger age than men [6].

Our patient experienced rapid progression. MRI showed sparseness of the pyramidal tract in diffusion tensor imaging, with restricted diffusion of the corticonuclear tract in diffusion-weighted imaging. CT showed a dot periventricular high-density shadow (with CT values in the range of 30–40 Hounsfield units) in the parietal lobe; consequently, we speculated that they might be calcifications. A previous report [7] found that such high-density shadows were calcifications. Our patient exhibited heavy involvement of the corpus callosum, which may be useful for a differential diagnosis [8]. The MRI showed foci with restricted diffusivity that persisted for 4.75 months [9]. Multiple paraventricular demyelinating lesions are common in patients with MS, and HDLS can mimic primary progressive MS. However, persistent diffusion-restricted lesions in the white matter might suggest CSF1R-related leukoencephalopathy because such lesions are rare in MS.

Diffusion-weighted imaging physiology-based neuroimaging techniques.

Diffusion-weighted imaging (DWI) assesses water diffusivity within intra- and extracellular spaces by means of apparent diffusion coefficient (ADC) measurements. Restricted diffusion can be seen in acute cerebral infarction, MS, brain tumor [10], neural intrahepatic inclusion disease (NIID) [11], carbon monoxide intoxication [12], etc. The duration of diffusion limitation varies with the nature of lesions. DWI in NIID showed high signals at the cerebral cortico-medullary junction, which persisted over 1 year and progressively increased [11]. Subcortical high DWI signals in NIID strongly correlate with pathological spongiotic changes of NIID [13]. The persistence of the diffusion-restricted lesions presumably reflect intramyelinic edema in regions of neurodegeneration [14]. As multiple conduction pathways with diffusion restriction are observed in our patient, we speculate it may be related to primary axonal degeneration and loss of myelin sheaths, which are the pathological features of ALSP. ADC, a measure reflecting the magnitude of free water mobility obtained by using DWI, can be reduced by the presence of increased cell membranes that typify hypercellular tumor [15, 16]. ADC can be reduced by other biophysical parameters, including increased viscosity; protein content, as occurring in abscess [17]; and movement of water from the extracellular to intracellular space as seen in ischemia or cytotoxic edema [18]. Acute cell necrosis with cytotoxic edema, as seen in acute ischemic infarction, did not develop in our patient. However, tissue injury caused by slowly progressive cytotoxic edema related to ALSP or some other unknown mechanism resulted in diffusion limitation in our patient.

The results of the MRI showed that there were particular hallmarks of CSF1R-related leukoencephalopathy, which could be used in a differential diagnosis. Unfortunately, our patient refused a brain biopsy. Confirmation of the genotype-phenotype relationship with CSF1R is needed through large, multicenter studies.

In conclusion, we identified a novel CSF1R mutation in our patient. The many lesions involving the pyramidal tract and white matter showed continuously restricted diffusion on brain imaging, gradually increasing with disease progression. In addition to white matter abnormalities, we propose that thinning of the corpus callosum, continuous diffusion-restricted lesions, and dot high-density lesions on CT are hallmarks of CSF1R-related leukoencephalopathy.

## Data Availability

Not applicable.

## Abbreviations

ALSP: Adult-onset leukoencephalopathy with axonal spheroids and pigmented glia
CSF1R: Colony-stimulating factor 1 receptor
MRI: Magnetic resonance imaging
HDLS: Hereditary diffuse leukoencephalopathy with spheroids
MS: Multiple sclerosis
CT: Computed tomography
DWI: Diffusion-weighted imaging
ADC: Apparent diffusion coefficient
NIID: Neural intrahepatic inclusion disease
